# The Prognostic Utility of ^18^F-Fluorodeoxyglucose Positron Emission Tomography-Computed Tomography-Based Analyses of Metabolic Response Rates in Newly Diagnosed Diffuse Large B Cell Lymphoma Patients

**DOI:** 10.3389/fonc.2022.772773

**Published:** 2022-05-23

**Authors:** Cong Li, Haifeng Yu, Xi Chen, Shuiyun Han, Shuailing Peng, Tao Lei, Haiyan Yang

**Affiliations:** ^1^Cancer Hospital of the University of Chinese Academy of Sciences (Zhejiang Cancer Hospital), Hangzhou, China; ^2^Institute of Cancer and Basic Medicine (IBMC), Chinese Academy of Sciences, Hangzhou, China

**Keywords:** diffuse large B cell lymphoma (DLBCL), interim 18 F-FDG PET, prognosis, RCHOP, treatment response

## Abstract

**Background:**

Roughly one third of diffuse large B cell lymphoma (DLBCL) patients experience relapsed or refractory disease, and their prognosis is unsatisfactory. It is thus important to identify patients who respond poorly to first-line treatment. Some studies have evaluated the prognostic value of interim PET-CT (iPET-CT) or end-of-treatment PET-CT (ePET-CT) in lymphoma patients, but there have been few studies exploring the prognostic value of metabolic response rates in the evaluation of DLBCL patients.

**Methods:**

Consecutive newly diagnosed DLBCL patients were screened from March 2013 to June 2020. Patients received at least four cycles of chemotherapy, and underwent baseline, iPET-CT and ePET-CT scanning. Kaplan-Meier survival curves with log-rank tests were employed to assess survival outcomes including overall survival (OS) and progression-free survival (PFS). Independent predictors of survival were identified through univariable and multivariable Cox regression analyses.

**Results:**

307 patients were evaluated. At the time of iPET-CT scanning, 250, 45, and 12 patients exhibited complete response (CR), partial response (PR), and stable disease (SD)/progressive disease (PD), respectively. The percentage of negative iPET-CT was 81.4% (250/307). Among 295 patients with ePET-CT, 262 (88.8%) achieved negativity and 33 (11.2%) exhibited positivity including 26 PR and 7 PD. The 2-year PFS and 2-year OS for patients with iPET-CT positivity were 50.7% and 76.5%, respectively, and were significantly shorter than those for patients with iPET-CT negativity (2-year PFS 82.7%, *p*<0.001; 2-year OS 94.2%, *p*<0.001). Patients with ePET-CT positivity had significant poorer 2-year PFS (48.1%) and 2-year OS (78.5%) compared with those ePET-CT negativity (2-year PFS 83.8%, *p*<0.001; 2-year OS 94.9%, *p*<0.001). The positivity rates on iPET-CT and ePET-CT evaluation were significantly higher in patients in the high/high-intermediate risk group compared with patients in the low/low-intermediate group. In a multivariable analysis, high/high-intermediate international prognostic index (IPI) and ePET-CT positivity were independently associated with poor PFS and OS.

**Conclusions:**

Our results suggest that the speed of metabolic response to treatment is of limited prognostic value in newly diagnosed DLBCL patients. Patients exhibiting PR at iPET-CT evaluation should carefully consider whether to change chemotherapy regimen.

## Introduction

Diffuse large B cell lymphoma (DLBCL) is the prevalent non-Hodgkin lymphoma subtype (1). Roughly 60% of patients with DLBCL can undergo successful curative first-line RCHOP (rituximab, cyclophosphamide, doxorubicin, vincristine, and prednisone) chemotherapy (2). Unfortunately, one third of patients still experience relapsed or refractory disease (3). Just 30-35% of these relapsed/refractory patients will undergo successful rescue by high-dose chemotherapy following autologous stem-cell transplantation (ASCT) (4). Therefore, further work is needed to efficiently identify patients that respond poorly to first-line therapy so that their chances of cure can be increased by early intensification.

^18^F-fluorodeoxyglucose positron emission tomography-computed tomography (^18^F-FDG PET-CT) is commonly employed in lymphoma patients for pretreatment staging, therapeutic efficacy evaluation, and transformation assessment (5). Positive end-of-treatment PET-CT (ePET-CT) scans are closely associated with residual/recurrent disease and with worse overall survival (OS) and progression-free survival (PFS) (6). However, the predictive role of the mid-treatment PET-CT remains controversial (7–9). As such, interim PET-CT-(iPET-CT)-guided therapy strategies in DLBCL patients have not been widely accepted to date.

In advanced mantle cell lymphoma (MCL), Jeon et al. suggested that the speed of metabolic response to treatment may be a powerful predictor of individual outcomes (10). It has been hypothesized that DLBCL patients who are rapid metabolic responders, as measured by reductions in the intensity of ^18^F-FDG uptake, are reflective of early tumor regression with a high likelihood of curative outcomes, whereas slow metabolic responders are more likely to relapse. To test this hypothesis, we conducted the present retrospective analysis to explore the prognostic value of metabolic response rate measured by iPET-CT and ePET-CT, indexed by the Deauville five-point scale, in a cohort of DLBCL patients undergoing treatment with a RCHOP-like regimen.

## Materials and Methods

### Patients and Study Design

Consecutive newly diagnosed DLBCL patients were screened from March 2013 to June 2020 at Zhejiang Cancer Hospital. The DLBCL diagnosis for these patients was confirmed *via* pathological review as performed by an independent experienced pathologist. Disease stage was judged according to the criteria of Lugano 2014 (11). First-line treatment consisted of at least four cycles of rituximab-containing anthracycline-based chemotherapy. Patients that completed fewer than four cycles were excluded. All patients underwent baseline whole-body PET-CT scans within four weeks before starting therapy, iPET-CT scans after four cycles of chemotherapy, and ePET-CT scans conducted within eight weeks after the completion of chemotherapy. Responses to chemotherapy were evaluated based upon the revised criteria published by Cheson et al. (12). The Deauville score (DS) was employed for measuring ^18^F-FDG-uptake in PET-CT (13). A DS 1 to 3 was defined as PET negativity. DS 4 or DS 5 were used to define PET positivity. After completion of first-line chemotherapy, all patients underwent regular follow-up CT scans every 3 months over the first two years, every 6 months for the next three years, and once a year from the sixth year onward. A retrospective analysis of data extracted from patient electronic medical records including demographic information, pathological features, treatment regimens, therapeutic responses to initial or salvage chemotherapy, and survival was performed. The Zhejiang Cancer Hospital ethics committee approved this study, which was consistent with the Declaration of Helsinki.

### Data Analysis

PFS was calculated from the start of first-line chemotherapy to the first recording of disease progression or disease relapse or death. OS was defined as the period from the start of first-line chemotherapy to the date of death from any cause or the last follow-up. Categorical variables are given as proportions and were analyzed with chi-squared tests and Fisher’s exact test. Continuous variables are given as medians and ranges. PFS and OS were calculated using the Kaplan–Meier survival method and log-rank tests. Univariable and multivariable Cox regression analyses were performed to determine the independent factors affecting PFS or OS. *P <*0.05 was the threshold of significance. To further explore exact survival differences, survival time distributions in four groups were compared pairwise. A Bonferroni corrected *p*-value was applied to the multifactorial logistic regression *p*–values to account for the multiple testing of six different comparisons (corrected α = 0.05/6 = 0.00833). Statistical analyses were performed with Statistical Package for Social Sciences (SPSS), version 24. Survival curves were drawn with GraphPad Prism 8.

## Results

### Clinical Characteristics

**Figure 1:**
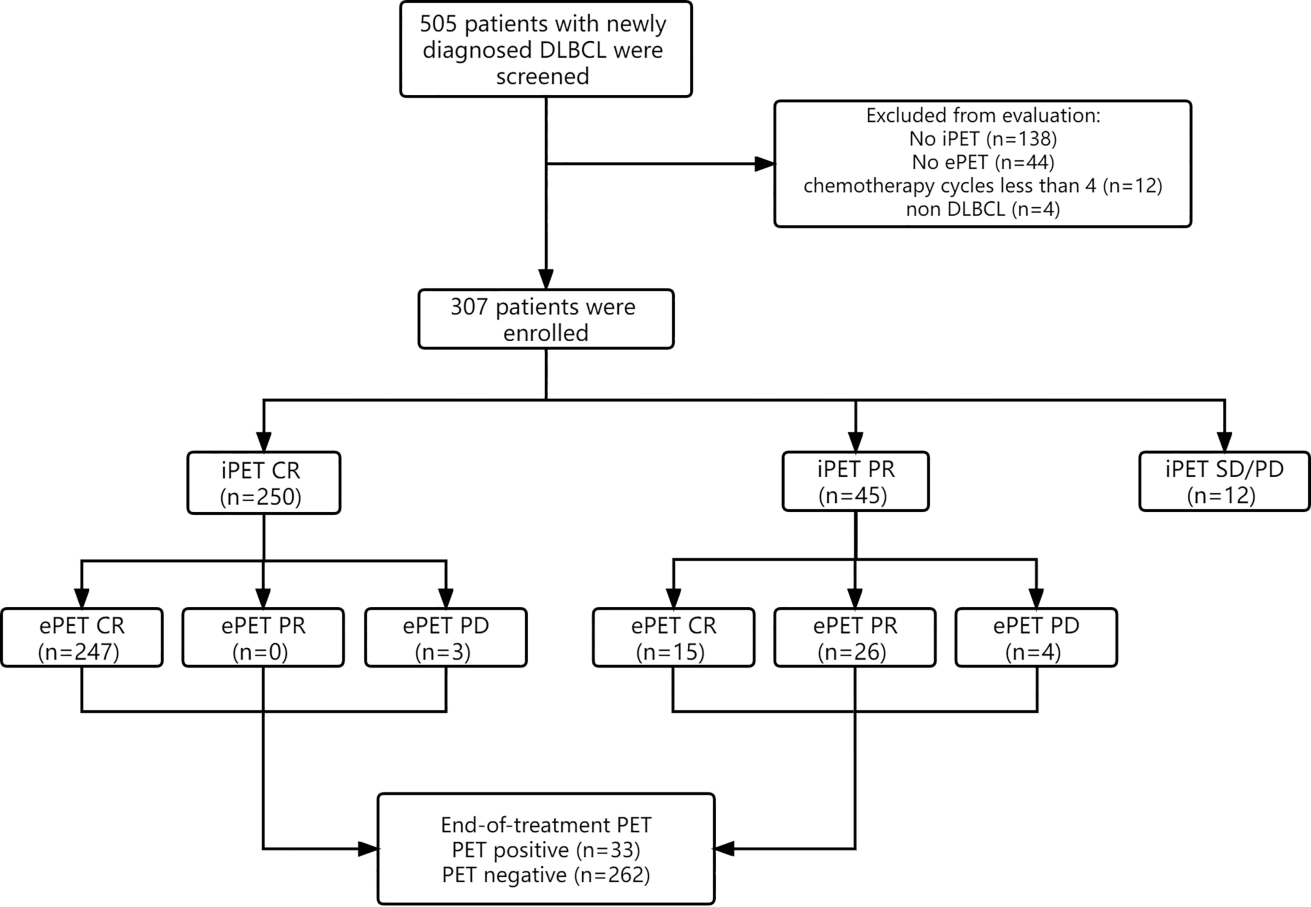
Flow diagram of 505 patients with newly diagnosed DLBCL. 198 patients were excluded from the study, resulting in 307 patients being analyzed. Response evaluation of iPET-CT and ePET-CT were showed.

Initially, 505 total patients diagnosed with DLBCL were identified, of whom 198 were excluded due to ambiguous diagnoses (n=4), fewer than 4 chemotherapy cycles (n=12), or a lack of available iPET-CT or ePET-CT data (n=182). Therefore, 307 patients were analyzed in this study (**Figure 1**). Patient baseline clinical characteristics are shown in **Table 1**.

**Table 1:** Baseline characteristics of DLBCL patients with frontline chemotherapy (n=307).

Characteristics	Number (%)
Age (in years)	Median 55 (range 15-84)
Gender Males Females	146 (47.6) 161 (52.4)
Pathological subtype GCB non-GCB	89 (29) 218 (71)
Ann-Arbor stage I-II III-IV	149 (48.5) 158 (51.5)
Bulky disease (>5cm) Yes No	78 (25.4) 229 (74.6)
ECOG performance status 0-1 2-3	270 (87.9) 37 (12.1)
Presence of B symptoms Yes No Bone marrow involvement Yes No	57 (18.6) 250 (81.4) 19 (6.2) 288 (93.8)
Elevated LDH Yes No	145 (47.2) 162 (52.8)
IPI 0-1 2-3 4-5	145 (47.2) 123 (40.1) 39 (12.7)
First-line chemotherapy regimen RCHOP REPOCH R2CHOP	240 (78.2) 63 (20.5) 4 (1.3)

GCB, germinal center B cell; ECOG, Eastern Cooperative Oncology Group; LDH, lactic dehydrogenase; IPI, international prognostic index; RCHOP, rituximab, cyclophosphamide, doxorubicin, vincristine and prednisone; REPOCH, rituximab, etoposide, cyclophosphamide, doxorubicin, vincristine and prednisone; R2CHOP, lenalidomide, rituximab, cyclophosphamide, doxorubicin, vincristine and prednisone.

### ^18^F-FDG PET-CT Treatment and Efficacy Evaluation

All 307 patients underwent initial pretreatment PET-CT and iPET-CT scanning (**Figure 1**). At iPET-CT evaluation, 250 patients achieved complete response (CR) and the proportion of patients with negative metabolic uptake was 81.4% (250/307). Moreover, 45 patients achieved partial response (PR), all of whom continued to complete prior chemotherapy regimens for at least 2 cycles, and 15 of them (33.3%) achieved CR at ePET-CT. Twenty-six patients maintained PR, while 4 patients ultimately exhibited progressive disease (PD). Additionally, 12 patients exhibited SD/PD at iPET-CT, of whom just 3 underwent biopsy and 2 were confirmed to have progressive disease. Of these 12 patients, 10 underwent second-line treatment, while one underwent palliative radiotherapy. The remaining patient did not receive any treatment, and died 5 months later.

At time of ePET-CT evaluation (n=295), 262 patients (88.8%) achieved CR and were considered as negative ePET-CT, whereas 33 patients (11.2%) exhibited ePET-CT positivity, including 26 patients with PR and 7 patients with PD. Among the 26 patients with PR at time of ePET-CT, 10 received second-line chemotherapy and 2 of them underwent subsequent autologous stem-cell transplantation (ASCT) with no evidence of disease. Eight patients received palliative radiotherapy for residual lesions without chemotherapy. Another 8 patients did not receive any treatment, and 7 of them were still alive. All 7 patients with PD at ePET-CT received salvage chemotherapy, but only 3 patients remained alive at last follow-up.

### PET-CT-Based Survival Outcomes

After a median follow-up of 45.1 months (range: 5.1 - 100 months), 81 patients (26.4%) experienced disease progression or relapse, and 36 patients (11.7%) were censored due to death. The 2-year PFS rate and 2-year OS rate for the whole cohort (n=307) were 76.6% (95% confidence interval (CI), 71.8 to 81.4%) and 91.0% (95% CI, 87.7 to 94.2%), respectively.

**Figure 2:**
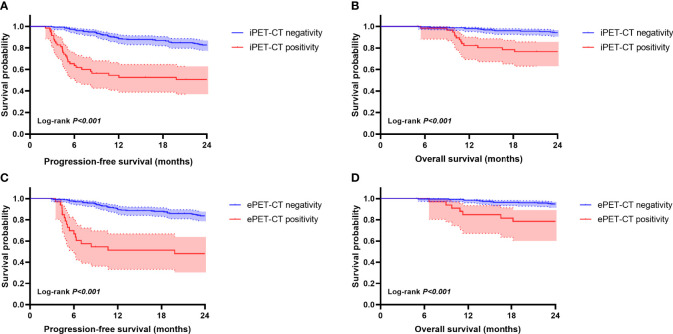
Kaplan-Meier survival curves according to interim PET-CT (iPET-CT) and end-of-treatment (ePET-CT). Progression-free survival (PFS) **(A)** and overall survival (OS) **(B)** according to iPET-CT evaluation. PFS **(C)** and OS **(D)** according to ePET-CT.

The iPET-CT and ePET-CT results for these patients were both significantly associated with survival outcomes (**Figure 2**). The 2-year PFS and 2-year OS for patients with iPET-CT positivity were 50.7% (95%CI, 37.6 to 63.8%) and 76.5% (95%CI, 65.3 to 87.7%), respectively, and were significantly shorter than those for patients with iPET-CT negativity (2-year PFS: 82.7% (95% CI, 78 to 87.4%), *p*<0.001; 2-year OS: 94.2% (95% CI, 91.3 to 97.1%), *p*<0.001). The survival outcomes for patients with SD/PD at iPET-CT were extremely poor, with median PFS and OS were only 3.2 months and 11.0 months, respectively. Similarly, patients with ePET-CT positivity had a significantly poorer 2-year PFS (48.1%, 95% CI, 30.9 to 65.3%) and 2-year OS (78.5%, 95% CI, 64.4 to 92.6%) rates compared with those of patients with ePET-CT negativity (2-year PFS: 83.8% (95% CI, 79.3 to 88.3%), *p*<0.001; 2-year OS: 94.9% (95% CI, 92.2 to 97.6%), *p*<0.001).

**Figure 3:**
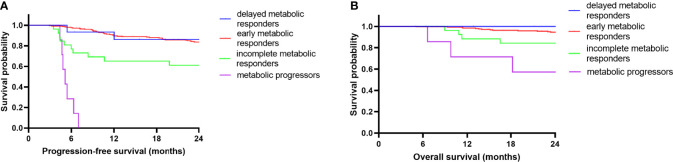
Kaplan-Meier survival curves according to serial changes in PET-CT response. PFS **(A)** and OS **(B)** according to early metabolic responders (n=247), delayed metabolic responders (n=15), incomplete metabolic responders (n=26) and metabolic progressors (n=7) during frontline RCHOP.

These results suggest that there are significant relationships between PET avidity at different follow-up time points and DLBCL patient survival. In light of these results, we conducted a further examination of the prognostic value of the speed of metabolic response. As patients with SD/PD at iPET-CT began undergoing second-line chemotherapy and lacked available ePET-CT scans, so they were excluded in this section. The remaining 295 patients were divided into the following 4 groups: EMR (early metabolic responders, iPET-CR+ePET-CR, n=247), DMR (delayed metabolic responders, iPET-PR+ePET-CR, n=15), IMR (incomplete metabolic responders, iPET-PR+ePET-PR, n=26), and MP (metabolic progressors, iPET-CR/PR+ePET-PD, n=7). The 2-year PFS rates were significantly different in these four groups (83.7%, 86.2%, 61.1%, and 0%, respectively; *p*<0.001). The 2-year OS rates were also significantly different in these four groups (94.6%, 100%, 84.3%, and 57.1%, respectively; *p*<0.001). The survival distribution of the four groups was compared in a pairwise manner. For 2-year PFS rate, there was a significant difference between MP and EMR (*p*<0.001), DMR (*p*<0.001), and IMR (*p*<0.001). There was also a difference between EMR and IMR (*p*=0.002). Between the other groups, no significant difference was found (*p*>0.0083). For 2-year OS, there was a difference between MP and EMR (*p*<0.001), and DMR (*p*=0.006). No significant difference was found between the other groups (*p*>0.0083). After Bonferroni correction, results showed a significant prognostic difference between MP and EMR/DMR. In **Figure 3**, a Kaplan-Meier plot for PFS and OS of the different groups of patients is shown.

The iPET-CT and ePET-CT positivity rates in different international prognostic index (IPI) risk groups were significantly different. Overall, 13.9% (29/209) patients with low/low-intermediate risk exhibited iPET-CT positivity, while 29.6% (29/98) patients with high/high-intermediate risk exhibited iPET-CT positivity (*p*=0.001). Moreover, 8.8% (18/205) patients with low/low-intermediate risk exhibited ePET-CT positivity, while 16.7% (15/90) patients with high/high-intermediate risk exhibited ePET-CT positivity (*p*=0.048). Additionally, bulky nodes (> 5 cm) and elevated serum C reactive protein (CRP) were more common in patients with positive iPET-CT (28.2% vs 15.7%, *p*=0.015; 26.1% vs 14.6%, *p*=0.013).

### Analysis of Prognostic Factors Associated With Patient Survival Outcomes

Factors including iPET-CT (positivity vs negativity), ePET-CT (positivity vs negativity) and IPI (high/high-intermediate vs low/low-intermediate) were analyzed in univariable and multivariable analysis for potential significance in terms of PFS and OS. In univariable analysis, positive iPET-CT, positive ePET-CT and high/high-intermediate IPI were all associated with inferior PFS and latter two factors were also associated with inferior OS. In multivariable analysis, positive ePET-CT and high/high-intermediate IPI were independent prognostic factors for poor PFS and OS (**Table 2**).

**Table 2:** Univariable and multivariable analysis of PFS and OS.

Survival	Univariable Cox Proportional hazard regression			Multivariable Cox proportional hazard regression		
	HR	95%CI	*p*	HR	95%CI	*p*
**PFS**						
iPET-CT positivity	2.4	1.4-4.0	0.002	0.4	0.1-1.3	0.129
ePET-CT positivity	4.1	2.4-7.0	<0.001	8.0	2.4-26.3	0.001
high/high-intermediate IPI	2.6	1.6-4.2	<0.001	2.3	1.4-3.8	0.001
**OS**						
iPET-CT positivity	2.2	0.9-5.0	0.057	0.5	0.1-1.8	0.272
ePET-CT positivity	3.9	1.8-8.6	0.001	5.6	1.5-20.2	0.009
high/high-intermediate IPI	4.8	2.3-10.4	<0.001	4.3	2.0-9.3	<0.001

PFS, progression-free survival; OS, overall survival; iPET-CT, interim positron emission tomography-computed tomography; ePET-CT, end-of-treatment positron emission tomography-computed tomography; IPI, international prognostic index.

## Discussion

In this cohort of 307 newly diagnosed DLBCL patients undergoing first-line rituximab-containing anthracycline-based chemotherapy treatment, the 2-year PFS and OS were 76.6% and 91.0%, respectively, in line with previous reports (14, 15).

Our study had several important findings. First, 81.4% (250/307) of patients achieved negative iPET-CT, of whom 98.8% (247/250) maintained CR after the completion of chemotherapy. These early metabolic responders had excellent survival outcomes, with a 2-year PFS of 83.7% and a 2-year OS of 94.6%. Second, only approximately 3.9% (12/307) of patients exhibited rapid disease progression and were considered as SD/PD at iPET-CT. The survival outcomes for these patients were poor, with median PFS and OS of just 3.2 months and 11.0 months, respectively. Third, although patients achieved negative iPET-CT findings, about 1.2% (3/250) of them still exhibited new metabolic lesions at ePET-CT. The survival outcomes of these 3 patients were poor. Intriguingly, among patients with PR at iPET-CT, 33.3% (15/45) of patients achieved CR at the end of chemotherapy. These delayed metabolic responders exhibited durable remission outcomes similar to those of early metabolic responders. Multivariable analyses further confirmed that ePET-CT positivity, but not iPET-CT positivity, was independently associated with patient prognosis. In summary, our study failed to confirm the hypothesis that there is a survival difference between early metabolic responders and delayed metabolic responders when evaluating DLBCL patients. These findings also indicate that the intensification of treatment regimens based upon iPET-CT positivity would likely expose many patients to the risk of unnecessary treatment.

A delayed metabolic response group has been noted in a few previous studies (10, 16, 17). A large, multinational, prospective study analyzed survival of patients with different metabolic response rates and found that 192 of 312 (62%) patients had negative iPET-CT and ePET-CT findings consistent with a rapid response, with a 2-year EFS of 97% and a 2-year OS of 97%. Moreover, 58 of 107 (54%) patients with positive iPET-CT findings achieved CR at ePET-CT, with an EFS of 86% and OS of 92%. The remaining 49 (16%) cases with positive iPET-CT and ePET-CT findings had a 2-year EFS of 35% and continuing relapses beyond 2 years. The delayed metabolic responders had approximately double the risk of 2-year relapse compared with early metabolic responders (18). Therefore, serial PET scans are important tools for the evaluation of lymphoma patients.

One possible explanation for delayed metabolic response is false-positive PET-CT results. Persistent ^18^F-FDG uptake can be indicative not only of residual lymphoma lesions but also of inflammatory reactions within necrotic tumor tissue (19). Such false positivity is more common in areas exposed to rituximab treatment (20). According to previous reports, the positive predictive value of iPET-CT ranged from 18% to 74% (16, 17, 21–23). This indicates that a single iPET-CT scan offers limited value as a means of identifying patients with poor outcomes. In addition, in patients exhibiting persistent FDG uptake in only one locus or the appearance of FDG uptake in a previously non-avid site, unrelated secondary neoplasms should be excluded (20). Particularly in cases of highly metabolically active PET-CT lesions within 1.5 cm in diameter, contrast-enhanced CT scans are important to exclude lymphoma lesions. Unfortunately, in this study, only a small number of patients with positive iPET-CT/ePET-CT findings underwent biopsy to confirm the presence of lymphoma and rule out potential secondary neoplasms.

Different criteria for the interpretation of PET results have certain limitations. The Deauville criteria, which is a visual assessment method, has been recommended by international guidelines and adopted for current clinical practice throughout the globe. In the present study, Deauville scores of 1-3 were considered as CR and PET negativity. But some patients with high Deauville scores could still achieve long survival time. As such, other semi-quantitative response assessment methods, including International Harmonization Project (24), Gallamini criteria (25), △SUV_max_ (26) and SUV_max-liver-based_ interpretation (27) can be used for response evaluation in patients with DLBCL.

There are certain limitations to this analysis that warrant consideration when interpreting these results. For one, this was a retrospective, single-center study without any prospective surveillance, and so these results may have been influenced by biases and other confounding variables. Secondly, this study excluded patients that only underwent CT scanning in order to focus on patients that had undergone iPET-CT and ePET-CT, thereby introducing selection bias. For survival analysis, we excluded patients with SD/PD at iPET-CT. The selection bias might influence the final survival outcome. Lastly, in most cases, disease progression was diagnosed in these patients based on imaging findings rather than biopsy results.

## Conclusions

Our results suggest that the speed of metabolic response to treatment offers limited prognostic value in newly diagnosed DLBCL patients. Patients exhibiting PR at iPET-CT evaluation should carefully consider whether to change chemotherapy regimen.

## Data Availability Statement

The original contributions presented in the study are included in the article/supplementary material. Further inquiries can be directed to the corresponding author.

## Ethics Statement

The studies involving human participants were reviewed and approved by The Zhejiang Cancer Hospital ethics committee. The patients/participants provided their written informed consent to participate in this study.

## Author Contributions

HYY, CL, and HFY participated in study design, evaluated the results, wrote the first and revised manuscript. CL performed the statistical analyses. SYH supervised patient care and collected data. CL, TL, HFY, XC, SP, and SH contributed to patient care and collected clinical information. All authors critically revised the manuscript and have approved the final version of the manuscript.

## Conflict of Interest

The authors declare that the research was conducted in the absence of any commercial or financial relationships that could be construed as a potential conflict of interest.

## Publisher’s Note

All claims expressed in this article are solely those of the authors and do not necessarily represent those of their affiliated organizations, or those of the publisher, the editors and the reviewers. Any product that may be evaluated in this article, or claim that may be made by its manufacturer, is not guaranteed or endorsed by the publisher.
